# Efficacy of Resveratrol in Experimental Subarachnoid Hemorrhage Animal Models: A Stratified Meta-Analysis

**DOI:** 10.3389/fphar.2022.905208

**Published:** 2022-06-29

**Authors:** Jiahe Tan, Rui Song, Siyue Luo, Wenqiao Fu, Yinrui Ma, Lian Zheng, Zhaohui He

**Affiliations:** ^1^ Department of Neurosurgery, The First Affiliated Hospital of Chongqing Medical University, Chongqing, China; ^2^ Key Laboratory of Molecular Biology for Infectious Diseases (Ministry of Education), Institute for Viral Hepatitis, Department of Infectious Diseases, The Second Affiliated Hospital of Chongqing Medical University, Chongqing, China; ^3^ Clinical Medicine, The Second Clinical College of Chongqing Medical University, Chongqing, China; ^4^ Department of Neurosurgery, The Fifth People’s Hospital of Chongqing Municipality, Chongqing, China

**Keywords:** resveratrol, subarachnoid hemorrhage, animal models, neuroprotection, meta-analysis

## Abstract

**Background:** Subarachnoid hemorrhage (SAH) is a serious neurosurgical emergency with extremely high morbidity and mortality rates. Resveratrol (RES), a natural polyphenolic phytoalexin, is broadly presented in a wide variety of plants. Previous research had reasonably revealed its neuroprotective effects on experimental SAH animal models to some extent. But the results were more controversial. Therefore, we conducted a meta-analysis to evaluate the evidence on the effectiveness of RES in improving outcomes in SAH animal models.

**Methods:** A systematic literature review was conducted in PubMed, EMBASE, and Web of Science databases to incorporate experimental control studies on the efficacy of RES on SAH models into our research. The standardized mean difference (SMD) was used to compare the brain water content (BWC) and neurological score (NS) between the treatment and control groups.

**Results:** Overall, 16 articles published from 2014 to 2022 met the inclusion criteria. The meta-analysis of BWC showed a significant difference in favor of RES treatment (SMD: −1.026; 95% CI: −1.380, −0.672; *p* = 0.000) with significant heterogeneity (Q = 84.97; I^2^ = 60.0%; *p* = 0.000). Further stratified analysis was performed for methodological differences, especially dosage, time of treatments, and time-point of outcome assessment. The meta-analysis of NS showed a significant difference in favor of RES treatment (SMD: 1.342; 95% CI: 1.089, 1.595; *p* = 0.000) with low heterogeneity (Q = 25.58; I^2^ = 17.9%; *p* = 0.223).

**Conclusion:** Generally, RES treatment showed an improvement in both pathological and behavioral outcomes in SAH animal models. The results of this study may provide a reference for preclinical and clinical studies in the future to some extent, with great significance for human health.

## Introduction

Subarachnoid hemorrhage (SAH), a disastrous neurosurgical disease due to its high morbidity and mortality rates, is mainly caused by a ruptured aneurysm or cerebrovascular malformation (Haut et al., 2006). The typical clinical manifestations of SAH are sudden onset of severe headache, nausea, vomiting, and meningeal irritation, with or without focal signs (van Gijn et al., 2007). Although the etiology of SAH can be eliminated by surgery in most patients, brain injury from SAH will still persist (Wong et al., 2012; Petridis et al., 2017). During the last decade, early brain injury (EBI) has been shown as an important factor leading to the poor prognosis of SAH in many studies (Pan et al., 2020; Li et al., 2021). EBI-induced neuronal damage will be permanent and lead to long-term neurologic impairment due to the poor regenerative capacity of the human brain (Rass and Helbok, 2019). Various effective therapies to reduce the loss of neurons after SAH are yet to be studied.

Resveratrol (RES), also known as 3–4′-5-trihydroxystilbene, is a natural polyphenolic phytoalexin that is widely present in a variety of plants such as *Vitis*, *Polygonum*, peanut, and *Veratrum* (Baur and Sinclair, 2006; Walle, 2011). *In vitro* and animal experiments have shown antioxidant, anti-inflammatory, anticancer, and cardiovascular protection effects of RES (Xia et al., 2017; Rauf et al., 2018; Meng et al., 2021). RES has been proved to have neuroprotective effects in cerebral ischemia, intracerebral hemorrhage, and neurodegenerative diseases in preclinical studies in recent years (Zhao et al., 2019; Liu et al., 2021; Su et al., 2021). In 2017–2018, our team found that RES could reduce brain water content (BWC) and improve neurological function in experimental SAH rats by alleviating neuronal apoptosis (Zhao et al., 2017a; Zhao et al., 2017b; Liang et al., 2019). During the last decade, there were studies that supported our results, while some did not (Li and Han, 2018; Zhou et al., 2021). Moreover, the methodological differences, especially dosage, timing of treatment, and time point of outcome assessment, were so divergent in each study that it was difficult to evaluate the overall therapeutic effect (Wan et al., 2019; Vellimana et al., 2020).

Up to date, no systematic review and meta-analysis have been conducted to evaluate the quality and synthesize evidence of preclinical studies on the effects of RES in SAH. Thus, the purpose of this study is to provide the preclinical evidence on the pathological and behavioral outcomes in RES-treated SAH animals. Furthermore, this preclinical meta-analysis may offer a reference for preclinical and clinical studies in the future on the RES treatment following SAH.

## Materials and Methods

### Literature Search

We conducted a comprehensive literature search in PubMed, EMBASE, and Web of Science databases to identify studies on the effect of RES in SAH animal models. Search terms included “subarachnoid hemorrhage” and “resveratrol” in Medical Subject Headings (MeSh) terms with their entry terms’ appropriate synonyms. The publication language was limited to English. The literature search period ended on May 22, 2022.

### Inclusion and Exclusion Criteria

We included the articles using the following criteria according to the evidence-based medicine literature retrieval format: 1) Population: experimental SAH animal models. 2) Interventions: RES was administered pre-SAH or post-SAH. 3) Comparisons: control animals were used. 4) Outcomes: therapeutic effects of RES were assessed using BWC or neurological score (NS). 5) Other criteria: experimental studies presented in original research articles and full text had to be available. The exclusion criteria were as follows: 1) Repetitive articles were excluded. 2) Co-treatments were performed. 3) Lack of end points of BWC or NS. Two reviewers (Tan and Song) independently performed title and abstract review and full-text examination according to the inclusion and exclusion criteria to determine the selected studies. Any disagreements were resolved by consensus with a third reviewer (Luo).

### Data Extraction

All of the data were extracted independently by two reviewers (Tan and Song). The information collected from each study included first author and publication year; species, gender, and age of animals; anesthetics used; method of SAH induction; intervention dosage (initial and total dosage); time point of treatment; number of animals per group; assessment time; pathological outcome (BWC) or functional outcome (NS); and methodological quality score. Any disagreements were resolved by consensus with a third reviewer (Luo). If the included studies used multiple experimental groups by different dosages, time points of treatment, or assessment time to compare against only one common control group, we divided these parallel groups equally into individual independent experiments and divided the size of the control group equally among treatment groups. If neurological score was performed at different time points, only the final time point was included. For every study, mean, standard deviation, or standard error of mean (SEM) of BWC and NS were extracted. For graphical data, GetData Graph Digitizer software (version 2.20) was used to measure values for mean and standard deviation from highly magnified images. Standard Deviations not directly reported were calculated by multiplying the reported SEM by the square root of the group size.

### Quality Assessment

Two reviewers (Tan and Song) independently evaluated the quality of each study according to the Collaborative Approach to Meta-Analysis and Review of Animal Data from Experimental Studies (CAMARADES) 10-item checklist (Macleod et al., 2004). One point was given for each of the following criteria: 1) peer-reviewed publication; 2) control of temperature; 3) random allocation to treatment or control group; 4) blinded induction of hemorrhage; 5) blinded assessment of outcome; 6) use of anesthetic without marked intrinsic neuroprotective activity; 7) animal model (aged, diabetic, or hypertensive); 8) sample size calculation; 9) compliance with animal welfare regulations; and (10) statement of potential conflict of interests. The studies’ quality was ranked as low (≤5 points) and high (>5 points). Any disagreements were resolved by consensus with a third reviewer (Luo).

### Statistical Analysis

Stata statistical software (version 16.0) was used to perform the meta-analysis. The standardized mean difference (SMD) was used to compare the RES’s effect on the BWC and NS between the treatment and control groups (Zeng et al., 2021). Heterogeneity across the studies was tested by calculating the I-squared (I^2^) statistic (Higgins et al., 2003). Whenever the I^2^ statistic was <50%, indicating low heterogeneity, then the fixed-effects model was used. On the contrary, whenever the I^2^ statistic was ≥50%, indicating high heterogeneity, then the random-effects model was used (Vetter, 2019). Sensitivity analysis was performed in which one study at a time was removed and the rest was analyzed to evaluate whether the results were affected by a single study. We conducted a stratified meta-analysis (Higgins and Thompson, 2002) to clarify the impact of methodological differences such as study quality, anesthetic drugs, methods to induce SAH, and especially dosage, timing of treatment, and time point of outcome assessment, using Review Manager software (version 5.3). Publication bias was detected using a funnel plot. Asymmetry was assessed using Egger’s test and the trim-and-fill method (Egger et al., 1997). *p* < 0.05 was considered statistically significant, and 95% confidence intervals (CI) were calculated for all results.

## Results

### Study Selection

**FIGURE 1:**
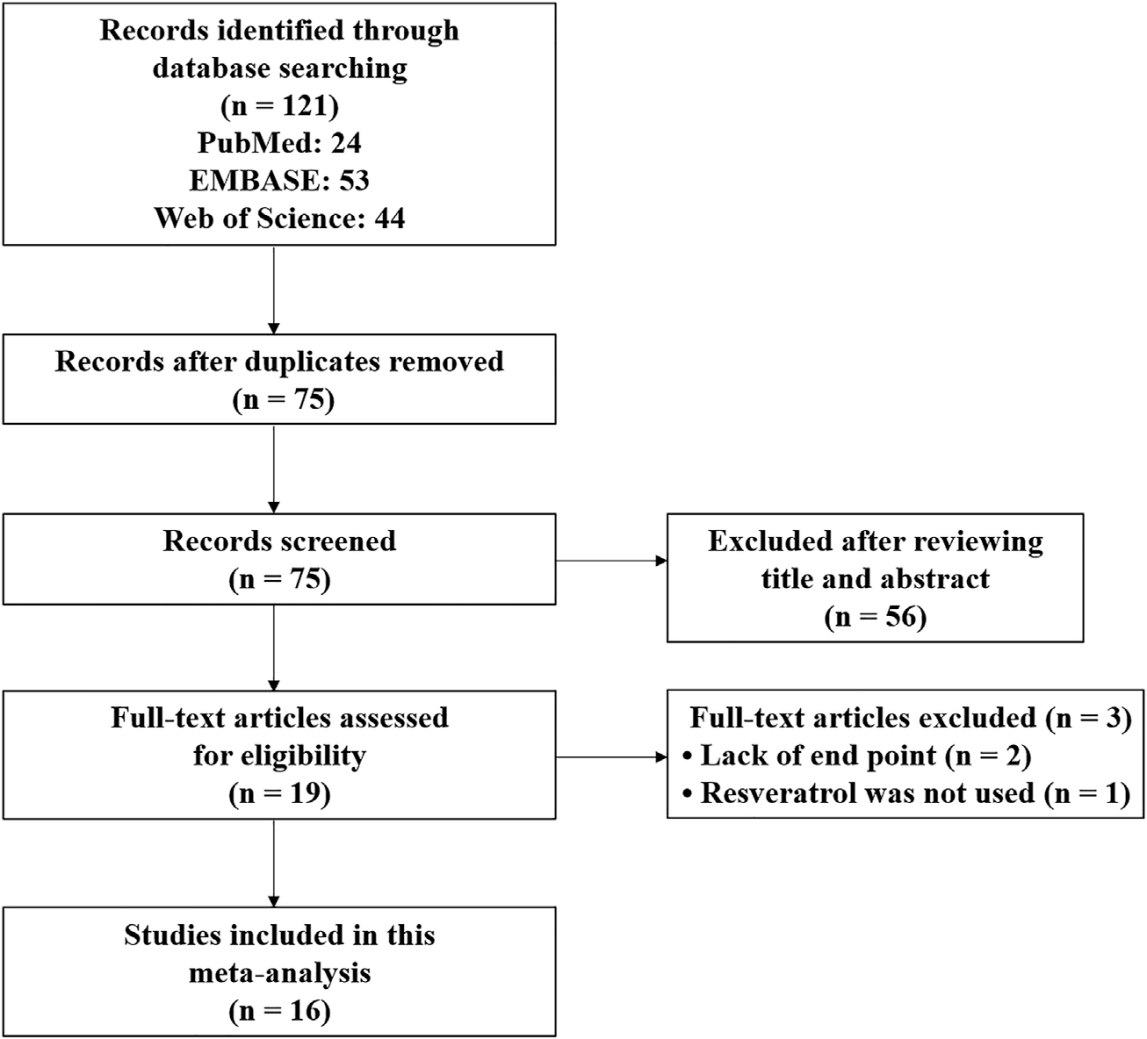
Flowchart of the literature search performed.

The systematic review and meta-analysis were conducted by following the Preferred Reporting Items for Systematic Reviews and Meta-Analysis (PRISMA) guidelines (Moher et al., 2009). Figure 1 showed the entire literature search process. After a comprehensive literature search in PubMed, EMBASE, and Web of Science databases, 121 records were identified. Then, 75 records remained for the title and abstract review after deleting duplicate records. After the title and abstract review, 19 articles were selected for full-text examination. Two were excluded because of missing end-point data for BWC and NS, and one was excluded because RES was not actually used in the experimental group. Finally, with the inclusion criteria, this study included 16 articles published from 2014 to 2022 (Shao et al., 2014; Zhang et al., 2016; Zhao et al., 2017a; Zhao et al., 2017b; Qian et al., 2017; Zhang et al., 2017; Guo et al., 2018; Li and Han, 2018; Liang et al., 2019; Wan et al., 2019; Xie et al., 2019; Vellimana et al., 2020; Diwan et al., 2021; Xiao et al., 2021; Zhou et al., 2021; Clarke et al., 2022). All articles were written in English.

### Study Characteristics

The characteristics of the included articles are shown in Table 1. There were 12 studies comprising 35 comparisons containing the data of BWC, while 16 studies comprising 22 comparisons containing the data of NS. These studies involved SD rats (*n* = 11), Wistar rats (*n* = 1), and C57 mice (*n* = 4). Almost all the studies used adult male animals except one that did not report the gender and age of animals. As for anesthesia, chloral hydrate (*n* = 3), pentobarbital (*n* = 5), and isoflurane (*n* = 7) were used, while one study did not report the anesthesia drug. Among them, chloral hydrate and pentobarbital were administered intraperitoneally (i.p.) and isoflurane was administered through inhalation. Further, endovascular perforation (*n* = 12) or autogenous blood injection (*n* = 4) was chosen to induce SAH in the model. RES was administered intraperitoneally in all the studies. The initial dosage of RES was 5–100 mg/kg, while the most frequent dosage was 60 mg/kg (*n* = 9). RES was given only once in 13 comparisons of 10 studies, while it was given repeatedly in 9 comparisons of 7 studies (one study used both ways of administration). The total dosage of RES was 5–240 mg/kg, while the most frequent dosage was 60 mg/kg (*n* = 6). The time of RES administration was divided into pre-SAH (*n* = 3) and post-SAH (*n* = 13). Among them, one study’s pre-SAH treatment group were administered multiple doses post-SAH. The maximum duration of treatment for RES was 48 h. Assessments were performed 24 (*n* = 9), 48 (*n* = 3), or 72 h (*n* = 5) after the induction of SAH (one study used two different time points to assess BWC and NS).

**TABLE 1:** Characteristics of the included studies.

Author, year	Animal, gender	Age	Anesthetic drug	Route	Method of SAH	Initial dosage	Total dosage	Treatment point	Treated (n)/Control (n)	Assessment time (h)	Outcome measure (direction)
Shao et al. (2014)	SD rats, NR	NR	NR	NR	Endovascular perforation	30 mg/kg	60 mg/kg	Immediately and 6 h post-SAH	10,10 10,10	24	BWC (lower is better) NS (higher is better)
Zhang et al. (2016)	SD rats, Male	Adult	Chloral hydrate	i.p.	Autogenous blood	60 mg/kg	120 mg/kg	2 and 12 h post-SAH	6,6 6,6	24	BWC (lower is better) NS (lower is better)
Zhao et al. (2017a)	SD rats, Male	Adult	Pentobarbital	i.p.	Endovascular perforation	60 mg/kg	60 mg/kg	1 h post-SAH	6,6 6,6	72	BWC (lower is better) NS (higher is better)
Zhao et al. (2017b)	SD rats, Male	Adult	Pentobarbital	i.p.	Endovascular perforation	60 mg/kg	60 mg/kg	1 h post-SAH	6,6 6,6	72	BWC (lower is better) NS (higher is better)
Qian et al. (2017)	SD rats, Male	Adult	Pentobarbital	i.p.	Endovascular perforation	100 mg/kg	100 mg/kg	48 h before SAH	6,6 6,6	24	BWC (lower is better) NS (higher is better)
Zhang et al. (2017)	SD rats, Male	Adult	Chloral hydrate	i.p.	Autogenous blood	60 mg/kg	120 mg/kg	2 and 12 h post-SAH	6,6 6,6	24	BWC (lower is better) NS (lower is better)
Guo et al. (2018)	SD rats, Male	Adult	Pentobarbital	i.p.	Endovascular perforation	60 mg/kg	60 mg/kg	Post-SAH	6,6 6,6	24	BWC (lower is better) NS (higher is better)
Wan et al. (2019) (1)	SD rats, Male	Adult	Isoflurane	Inhalation	Endovascular perforation	10 mg/kg	10 mg/kg	1 h post-SAH	6,2	24	NS (higher is better)
Wan et al. (2019) (2)	SD rats, Male	Adult	Isoflurane	Inhalation	Endovascular perforation	30 mg/kg	30 mg/kg	1 h post-SAH	6,2 6,6	24	BWC (lower is better) NS (higher is better)
Wan et al. (2019) (3)	SD rats, Male	Adult	Isoflurane	Inhalation	Endovascular perforation	90 mg/kg	90 mg/kg	1 h post-SAH	6,2	24	NS (higher is better)
Wan et al. (2019) (4)	SD rats, Male	Adult	Isoflurane	Inhalation	Endovascular perforation	30 mg/kg	30 mg/kg	1 h post-SAH	6,6 6,6	24	BWC (lower is better) NS (higher is better)
Liang et al. (2019)	SD rats, Male	Adult	Chloral hydrate	i.p.	Endovascular perforation	5 mg/kg	5 mg/kg	1 h post-SAH	6,6 6,6	24	BWC (lower is better) NS (higher is better)
Li and Han (2018)	SD rats, Male	Adult	Pentobarbital	i.p.	Endovascular perforation	60 mg/kg	60 mg/kg	2 h post-SAH	6,6 6,6	72	BWC (lower is better) NS (higher is better)
Xie et al. (2019)	Wistar rats, Male	Adult	Isoflurane	Inhalation	Autogenous blood	60 mg/kg	240 mg/kg	2, 6, 24, and 46 h post-SAH	6,6 6,6	48	BWC (lower is better) NS (higher is better)
Vellimana et al. (2020)(1)	C57 mice, Male	Adult	Isoflurane	Inhalation	Endovascular perforation	20 mg/kg	20 mg/kg	1 h before SAH	14,10	48	NS (higher is better)
Vellimana et al. (2020)(2)	C57 mice, Male	Adult	Isoflurane	Inhalation	Endovascular perforation	20 mg/kg	100 mg/kg	1 h before SAH (every 12 h for 2 days)	12,10	48	NS (higher is better)
Vellimana et al. (2020)(3)	C57 mice, Male	Adult	Isoflurane	Inhalation	Endovascular perforation	20 mg/kg	100 mg/kg	1 h before SAH (every 12 h for 2 days)	16,7	48	NS (higher is better)
Vellimana et al. (2020)(4)	C57 mice, Male	Adult	Isoflurane	Inhalation	Endovascular perforation	10 mg/kg	50 mg/kg	1 h before SAH (every 12 h for 2 days)	9,7	48	NS (higher is better)
Zhou et al. (2021)(1)	SD rats, Male	Adult	Isoflurane	Inhalation	Autogenous blood	60 mg/kg	120 mg/kg	2 and 24 h post-SAH	6,6	72	NS (higher is better)
Zhou et al. (2021)(2)	SD rats, Male	Adult	Isoflurane	Inhalation	Autogenous blood	60 mg/kg	120 mg/kg	2 and 24 h post-SAH	6,6	24	BWC (lower is better)
Diwan et al. (2021)	C57 mice, Male	Adult	Isoflurane	Inhalation	Endovascular perforation	6 mg/kg	30 mg/kg	3 h post-SAH (every 12 h for 2 days)	12,11	48	NS (higher is better)
Xiao et al. (2021)	C57 mice, Male	Adult	Isoflurane	Inhalation	Endovascular perforation	60 mg/kg	60 mg/kg	1 h before SAH	6,6	24	NS (higher is better)
Clarke et al. (2022)	C57 mice, Male	Adult	Isoflurane	Inhalation	Endovascular perforation	6 mg/kg	6 mg/kg	3 h post-SAH	16,29	72	NS (higher is better)

Lower is better: better prognosis with lower value; higher is better: better prognosis with higher value.

### Quality Assessment

The details of the quality index are presented in Table 2. The quality scores ranged from 2 to 7, with a mean value of 5.38. All included studies were peer-reviewed publications. Ten studies reported control of temperature. Nine studies reported random allocation to treatment or control group. Nine studies reported blinded assessment of outcome. All except one study reported the use of anesthetic without marked intrinsic neuroprotective activity and compliance with animal welfare regulations. None of them used blinded induction of SAH, used animals with relevant comorbidities, or reported a sample size calculation. Twelve studies declared no potential conflict of interests.

**TABLE 2:** Quality scores of 16 included studies.

Study, year	(1)	(2)	(3)	(4)	(5)	(6)	(7)	(8)	(9)	(10)	Total
Shao et al. (2014)	★									★	2
Zhang et al. (2016)	★	★	★		★	★			★	★	7
Zhao et al. (2017a)	★	★				★			★	★	5
Zhao et al. (2017b)	★	★			★	★			★	★	6
Qian et al. (2017)	★	★	★		★	★			★		6
Zhang et al. (2017)	★		★			★			★	★	5
Guo et al. (2018)	★	★	★			★			★	★	6
Wan et al. (2019)	★	★				★			★	★	5
Liang et al. (2019)	★	★	★		★	★			★	★	7
Li and Han (2018)	★	★	★		★	★			★	★	7
Xie et al. (2019)	★		★		★	★			★	★	6
Vellimana et al. (2020)	★		★		★	★			★		5
Zhou et al. (2021)	★	★	★		★	★			★	★	7
Diwan et al. (2021)	★					★			★		3
Xiao et al. (2021)	★					★			★		3
Clarke et al. (2022)	★	★			★	★			★	★	6

### Overall Response

**FIGURE 2:**
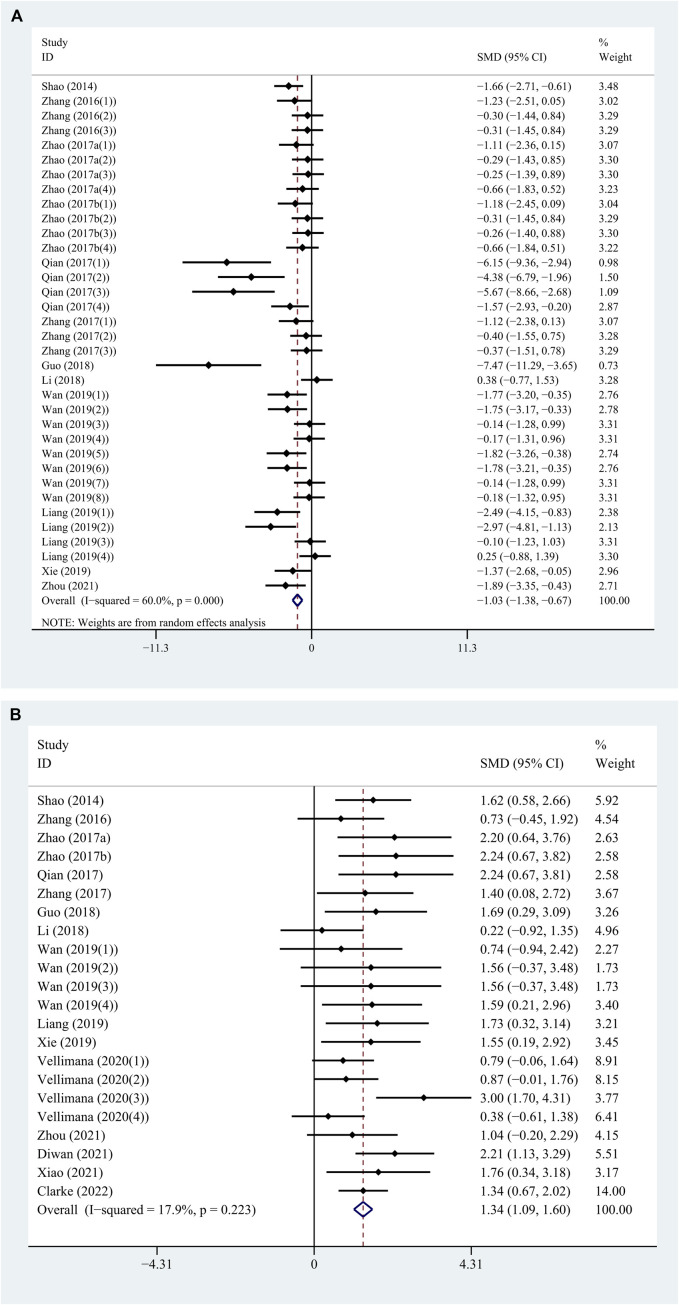
(continued).

On one hand, the treatment with RES revealed a significant reduction in BWC by an SMD of −1.026 (95% CI: −1.380, −0.672; *p* = 0.000, 12 studies, 35 comparisons, Figure 2A), with statistically significant heterogeneity (Q = 84.97; I^2^ = 60.0%; *p* = 0.000). So, we performed further stratified analysis from methodological differences, especially dosage, time of treatment, and time point of outcome assessment. On the other hand, the treatment with RES had a favorable effect on NS outcome by an SMD of 1.342 (95% CI: 1.089, 1.595; *p* = 0.000, 16 studies, 22 comparisons, Figure 2B), with low heterogeneity (Q = 25.58; I^2^ = 17.9%; *p* = 0.223). Therefore, further stratified analysis was not performed.

### Sensitivity Analysis

**FIGURE 3:**
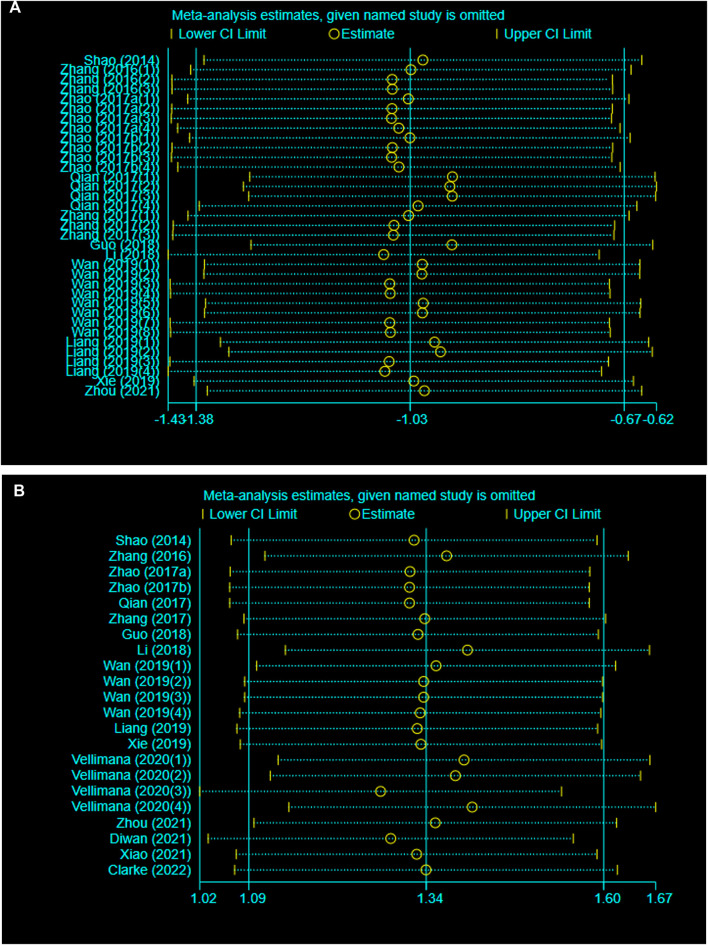
(continued).

A sensitivity analysis was conducted to evaluate the stability of our results by sequential omission of each study. Neither BWC nor NS was significantly affected by any study for the pooled SMD (Figures 3A, B).

### Publication Bias

**FIGURE 4:**
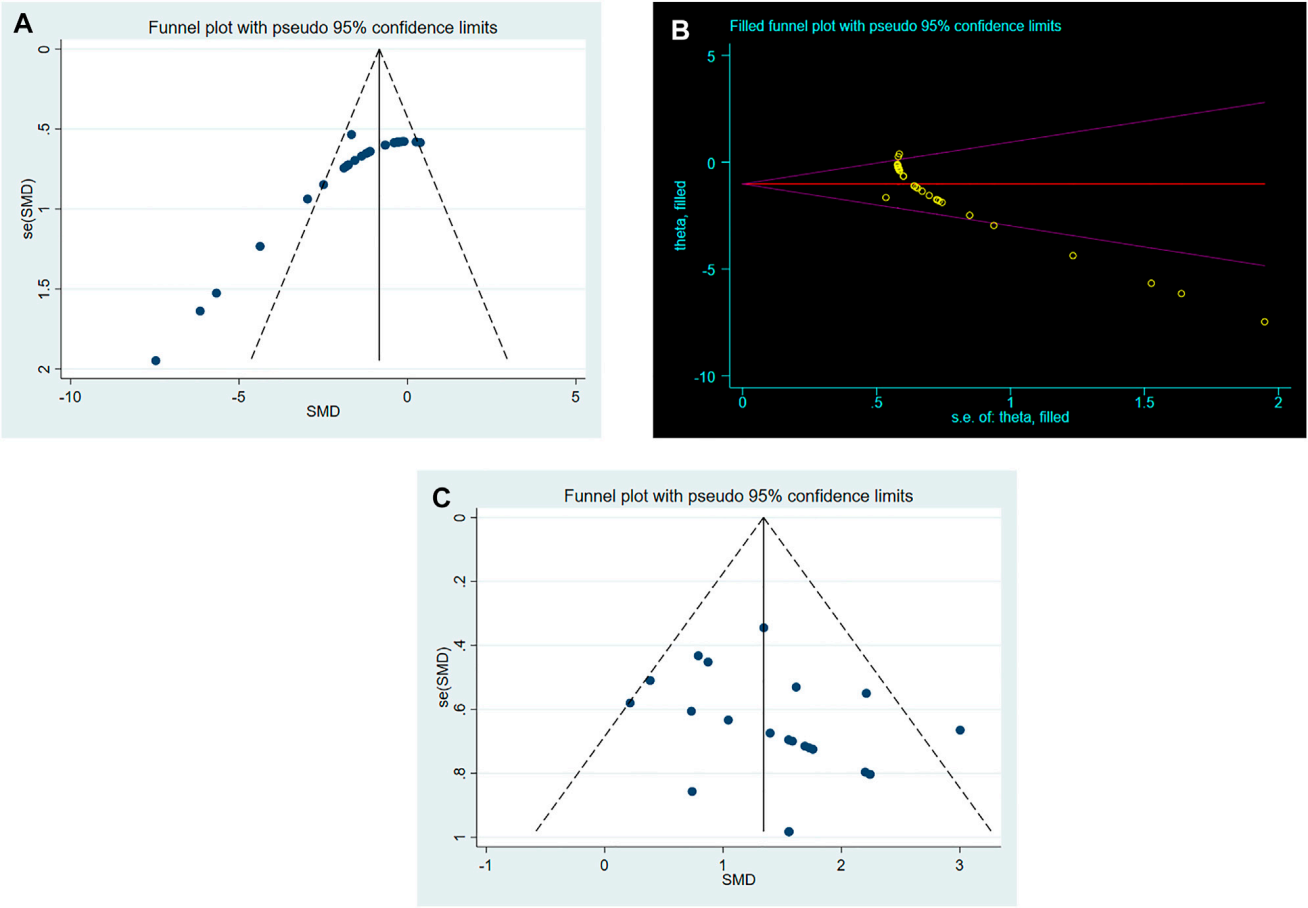
**(A)**. Funnel plots for RES treatment of BWC. **(B)**. Trim-and-fill analysis of RES treatment of BWC. **(C)**. Funnel plots for RES treatment of NS.

As shown in Figure 4A, conspicuous publication bias for BWC was suggested by visual inspection of the funnel plot. Then it was confirmed by the result of Egger’s test (*p* = 0.000). Under the circumstances, to estimate the missing studies and recalculate effect estimates, the trim-and-fill analysis was conducted. The result was consistent (SMD: −1.026; 95% CI: −1.380, −0.672; *p* = 0.000), indicating no “missing” studies (Figure 4B). As shown in Figure 4C, the funnel plot for the comparison of NS was approximately symmetrical. Besides, no significant publication bias was confirmed by the result of Egger’s test (*p* = 0.085).

### Stratified Analysis

As shown in Table 3, we performed a stratified analysis of BWC. First, we stratified the data by study quality. Studies were classified into high and low quality, and there was no significant difference in estimated effect size between them (X^2^ = 2.98, *p* = 0.08, Supplementary Figure S1). Concerning the anesthesia drugs, there was no significant difference in estimated effect size between the three different anesthetics used in the studies (X^2^ = 1.98, *p* = 0.39, Supplementary Figure S2). Although two different SAH induction methods were used in the studies, there was still no significant difference in estimated effect size between them (X^2^ = 1.26, *p* = 0.26, Supplementary Figure S3). The total dosage of RES was 5–240 mg/kg, and the stratified analysis showed no significant difference in the estimated effect size (X^2^ = 8.68, *p* = 0.12, Supplementary Figure S4) among them. Further, the stratified analysis showed no significant difference between single-dose and multiple-dose groups (X^2^ = 0.44, *p* = 0.51, Supplementary Figure S5). In addition, pre- and post-SAH treatments showed a significant difference in the estimates of effect size (X^2^ = 7.41, *p* = 0.006, Supplementary Figure S6). Finally, the time points of outcome assessment, which included 24, 48, and 72 h, showed a significant difference in the estimates of effect size (X^2^ = 8.03, *p* = 0.02, Supplementary Figure S7).

**TABLE 3:** Stratified meta-analysis of brain water content.

Subgroup	No. of studies	SMD (95%CI)	Heterogeneity test			*Χ* ^*2*^	*P*
			Q	*I* ^*2*^	*p*		
1 Study quality							
High	19	−1.40 (−2.04, −0.77)	66.52	73%	0.00001		
Low	16	−0.77 (−1.10, −0.44)	17.93	16%	0.00001		
						2.98	0.08
2 Anesthetic drug							
Chloral hydrate	10	−0.73 (−1.27, −0.19)	16.62	46%	0.05		
Pentobarbital	14	−1.38 (−2.14, −0.62)	50.18	74%	0.00001		
Isoflurane	10	−1.00 (−1.51, −0.48)	14.62	38%	0.1		
						1.89	0.39
3 Method to induce SAH							
Endovascular perforation	27	−1.14 (−1.60, −0.69)	78.86	67%	0.00001		
Autogenous blood	8	−0.78 (−1.21, −0.35)	6.02	0%	0.54		
						1.26	0.26
4 Total dosage of RES							
5 mg/kg	4	−1.19 (−2.70, 0.32)	14.06	79%	0.003		
30 mg/kg	8	−0.86 (−1.45, −0.27)	12.15	42%	0.10		
60 mg/kg	11	−0.73 (−1.28, −0.18)	21.79	54%	0.02		
100 mg/kg	4	−4.18 (−6.58, −1.77)	12.24	75%	0.007		
120 mg/kg	7	−0.71 (−1.17, −0.25)	5.17	0%	0.52		
240 mg/kg	1	−1.37 (−2.68, −0.05)					
						8.68	0.12
5 Dose administration of RES							
Single dose	26	−1.12 (−1.59, −0.65)	76.45	67%	0.00001		
Multiple doses	9	−0.91 (−1.32, −0.50)	8.33	4%	0.00001		
						0.44	0.51
6 Time administration of RES							
Pre-SAH treatment	4	−4.18 (−6.58, −1.77)	12.24	75%	0.007		
Post-SAH treatment	31	−0.81 (−1.11, −0.50)	54.28	45%	0.004		
						7.41	0.006
7 Time point of outcome assessment							
24 h	25	−1.32 (−1.81, −0.83)	74.29	68%	0.00001		
48 h	1	−1.37 (−2.68, −0.05)					
72 h	9	−0.45 (−0.84, −0.06)	4.98	0%	0.76		
						8.03	0.02

## Discussion

### Summary of Evidence

Over the past few decades, research studies have been conducted to explore the potential of various natural compounds to treat neurological diseases, while RES became a popular choice for many studies (Poulose et al., 2015). RES, a promising candidate for neuroprotection, has been proved to improve prognosis in traumatic brain injury and ischemic stroke animal models (Lopez et al., 2015; Liu et al., 2021). To the best of our knowledge, no meta-analysis has been conducted to evaluate the efficacy of RES in SAH animal models, and our study fills this gap. Our study demonstrated that RES helped in reducing BWC (SMD: −1.026; 95% CI: −1.380, −0.672; *p* = 0.000) and improving NS (SMD: 1.342; 95% CI: 1.089, 1.595; *p* = 0.000) in SAH animal models. The results of this meta-analysis in preclinical studies suggest that RES may have a potential application value in providing a neuroprotective effect in clinical SAH patients.

### Possible Mechanisms

RES has attracted much attention in preclinical studies of neurological diseases, not only because of its good biocompatibility but also its ability to quickly cross the blood–brain barrier via simple diffusion (Xie et al., 2019). On one hand, RES exhibited several functions including mitochondrial protective effect, enhancement of autophagy, inhibiting proinflammatory cytokine production, and anti-inflammatory and anti-apoptotic effects (Li and Han, 2018; Zhou et al., 2021). These effects of RES may ameliorate EBI in SAH. On the other hand, via reducing oxidative stress and increasing nitric oxide availability, RES protected cerebrovascular endothelial cell function (Schmitt and Dirsch, 2009; Chang et al., 2011). These effects of RES may relieve cerebral vasospasm and have an improvement on delayed cerebral ischemia (DCI) in SAH. Therefore, we hold the opinion that RES is a promising neuroprotective candidate in the future, which deserves more research in the treatment of SAH.

### Interpretation of Stratified Analysis

In this meta-analysis, RES had significant neuroprotective effects in reducing BWC and improving NS. But the heterogeneity among BWC groups was statistically significant (Q = 84.97; I^2^ = 60.0%; *p* = 0.000). Thus, the next step of stratified analysis from methodological differences, especially dosage, time of treatment, and time point of outcome assessment, was performed.

### Study Quality

Whether the quality of research could influence the estimation of effect size was debated since the beginning of this meta-analysis. Some meta-analyses suggested that the quality of research has a significant impact on the outcome (Macleod et al., 2004; Macleod et al., 2005), while others had a disagreement, denoted different qualities had no significant difference (Li et al., 2014; Cui et al., 2015). According to the results of our meta-analysis, studies with high quality tended to show higher efficacy than studies with low quality, but without significant difference. Although this trend of high quality versus high effect size is logical, more research will be needed to get a more definitive result. Besides, we suggest that future studies refer to the CAMARADES checklists (Macleod et al., 2004) when designing to ensure high quality.

### Anesthetic Drug

To date, no systematic review has been conducted to discuss the effects of anesthetics use in SAH models. Although phenobarbital achieved the best effect, the final result indicated no significant difference of effect size between the three groups. It has been reported that the three anesthetics might have potential neuroprotective roles (Macleod et al., 2005; Liu et al., 2015; Jiang et al., 2017), but their efficacy remained uncertain in SAH models. The inhibition of NMDA excitatory receptors and GABA receptors might be the specific mechanism for these anesthetics (Muir, 2010; Olsen and Li, 2011). With the improvement of experimental technology and the introduction of high-tech equipment, traditional intraperitoneal anesthesia has gradually been replaced by inhalation anesthesia for animals (Han et al., 2021). Besides, inhalation anesthesia was also widely used in human surgery (Papazian et al., 2016). Therefore, future studies need to focus on finding an inhalation anesthetic with minimal impact on the pathophysiologic process of SAH.

### Method to Induce SAH

As for the most commonly used models in SAH animals, both the endovascular perforation and the autogenous blood injection enjoy great fame (Marbacher et al., 2018). But, unfortunately, the physiological conditions of human SAH are so complicated that no animal model at the moment could simulate it. The result of our stratified analysis showed no significant difference of effect size among the two methods. The injection site and volume of blood were different in the autogenous blood injection model, while the sutures used for puncture were different in the endovascular perforation model (Marbacher, 2016). However, it is still necessary to use these two methods in animals to simulate the physiological conditions of human SAH to some extent, in order to continue the study of SAH. More suitable SAH models need to be found in the future.

### Dosage and Time of RES

Above all, the great variations in the total dosage, frequency of intervention, and time of administration made it hard to draw conclusions. The total dosage of RES was 5–240 mg/kg. Our result suggested that 100 mg/kg of RES had a better curative effect than other dosages, but the dosage ranges were too large to make reliable assessments, with no significant difference between these groups. Actually, only one author used the 100 mg/kg dosage in 4 comparisons, making the evidence for optimal dosage even weaker. After oral administration, the elimination half-life of RES was 9.2 h in human. While after intravenous injection, the elimination half-life of RES was 11.4 h in human (Baur and Sinclair, 2006). In SD rats, the time was reduced to 4.8 h after intravenous injection (Ng et al., 2014). Therefore, *in vivo*, multiple-dose therapy of RES may overcome the problem of short duration of action, achieving higher efficacy than single-dose therapy in SAH. But beyond our expectation, the result showed that the effect size of studies using multiple doses was not significantly different from those using a single dose. Small sample sizes and different total dosages may have an effect on the results in individual studies. In addition, significant differences in estimates of effect size were found between pre- and post-SAH treatment groups. The time of RES therapy could theoretically target different mechanisms. Early treatment could increase nitric oxide availability, reduce oxidative stress, and inhibit apoptosis (Li and Han, 2018; Zhou et al., 2021). Late treatment could reduce proinflammatory cytokine production and inhibit neuroinflammation (Schmitt and Dirsch, 2009; Chang et al., 2011). To sum up, pre-SAH treatment may be more neuroprotective in reducing EBI, which occurred rapidly after SAH (Rass and Helbok, 2019). With significant heterogeneity still existing in both groups, formulating relevant standards for the total dosage, frequency of intervention, and time administration will be particularly important in the future.

### Time Point of Outcome Assessment

BWC was measured at three time points of 24, 48, and 72 h after SAH, according to the choice of researchers. Statistically significant differences were found among these three groups, while the 48-h group has higher efficacy than other two groups. Although the difference was statistically significant, there was only one study that chose the 48-h time point. Too few studies might lead to an overestimation of the efficacy of RES. Besides, many studies suggested that 24 h after SAH is the optimum time point to complete the pathological and functional detection of EBI in animals (Lin et al., 2021). Therefore, normalizing the selection of the observation point needs to be performed in future studies.

### Advantages and Limitations

Great efforts have been made to get objective results in our meta-analysis. First, for providing the most complete evidence for RES treatment, our team attempted to review most of the reports associated with this field. Then, in order to reduce potential publication bias of the included studies, two practiced reviewers independently evaluated and extracted all the data and dealt with disagreements reasonably. Finally, our meta-analysis showed that RES treatment resulted in an improvement in both pathological and behavioral outcomes in SAH animal models. Sensitivity analysis confirmed stable results of BWC and NS, while stratified analysis detected heterogeneity in the result of BWC from methodological differences. To some extent, the results suggested the neuroprotective role of RES treatment, indicating RES may serve as a new therapeutic strategy for clinical SAH patients.

The present research achieved positive outcomes; however, some limitations still exist: 1) Our research only included data published in English. Although the search strategy was detailed, there is still the possibility that some published studies were missed. Some negative results were less likely to be published. Therefore, this meta-analysis may have exaggerated the effect size. 2) Our study included a relatively small number of published studies with highly significant heterogeneity, with a number of influence factors such as study quality, anesthetic drug, method to induce SAH, total dosage, dose administration, time of administration of treatment, and time point of outcome assessment. Although a further stratified analysis was conducted, the differences between most subgroups were still not significant. These results may be related to insufficient sample size and a lack of statistical capability. Therefore, sufficient evidence needs to be provided in the studies with large sample sizes in the future. 3) Specific SAH animal models with comorbidities such as hypertension or diabetes were not mentioned in the included articles, so it was not possible for our study to evaluate the efficacy of RES in these situations. 4) There was no study in this meta-analysis that evaluated the potential side effects of RES treatment in SAH. High dosage of RES (1,000 mg/kg/day) has been reported to cause hepatic and renal toxicity (Crowell et al., 2004; Rocha et al., 2009). It was not possible for our study to evaluate the safety of RES treatment either. Thus, for the clinical translation of RES treatment, significant work still has to be done.

## Conclusion

Our systematic review and meta-analysis revealed that RES treatment showed an improvement in both pathological and behavioral outcomes in SAH animal models. Limitations of the experimental design and methodological quality should be considered when interpreting the results. The results of this study may provide a reference for preclinical and clinical studies in the future, to some extent, with great significance for human health.

## Data Availability

The original contributions presented in the study are included in the article/Supplementary Material; further inquiries can be directed to the corresponding authors.

## Supplementary Material

Supplementary Figure S1 Stratified analysis by study quality.

Supplementary Figure S2 Stratified analysis by anesthetic drug.

Supplementary Figure S3 Stratified analysis by method to induce SAH.

Supplementary Figure S4 Stratified analysis by total dosage of RES.

Supplementary Figure S5 Stratified analysis by dose administration of RES.

Supplementary Figure S6 Stratified analysis by time administration of RES.

Supplementary Figure S7 Stratified analysis by time point of outcome assessment.

## Abbreviations

BWC, brain water content; CAMARADES, Collaborative Approach to Meta-Analysis and Review of Animal Data from Experimental Studies; DCI, delayed cerebral ischemia; EBI, early brain injury; SAH, subarachnoid hemorrhage; RES, resveratrol; NS, neurobehavioral score; SD, Sprague–Dawley; i.p., intraperitoneal; MeSh, Medical Subject Headings; SMD, standardized mean difference; SEM, standard error of mean; I2, I-squared; CI, confidence intervals; PRISMA, Preferred Reporting Items for Systematic Reviews and Meta-Analysis; NR, not reported.
