# Supplementary figures and images for: Efficacy of Resveratrol in Experimental Subarachnoid Hemorrhage Animal Models: A Stratified Meta-Analysis
Source: Front Pharmacol. 2022 Jun 29;13:905208. doi: 10.3389/fphar.2022.905208 (PMC9277348; doi:10.3389/fphar.2022.905208)

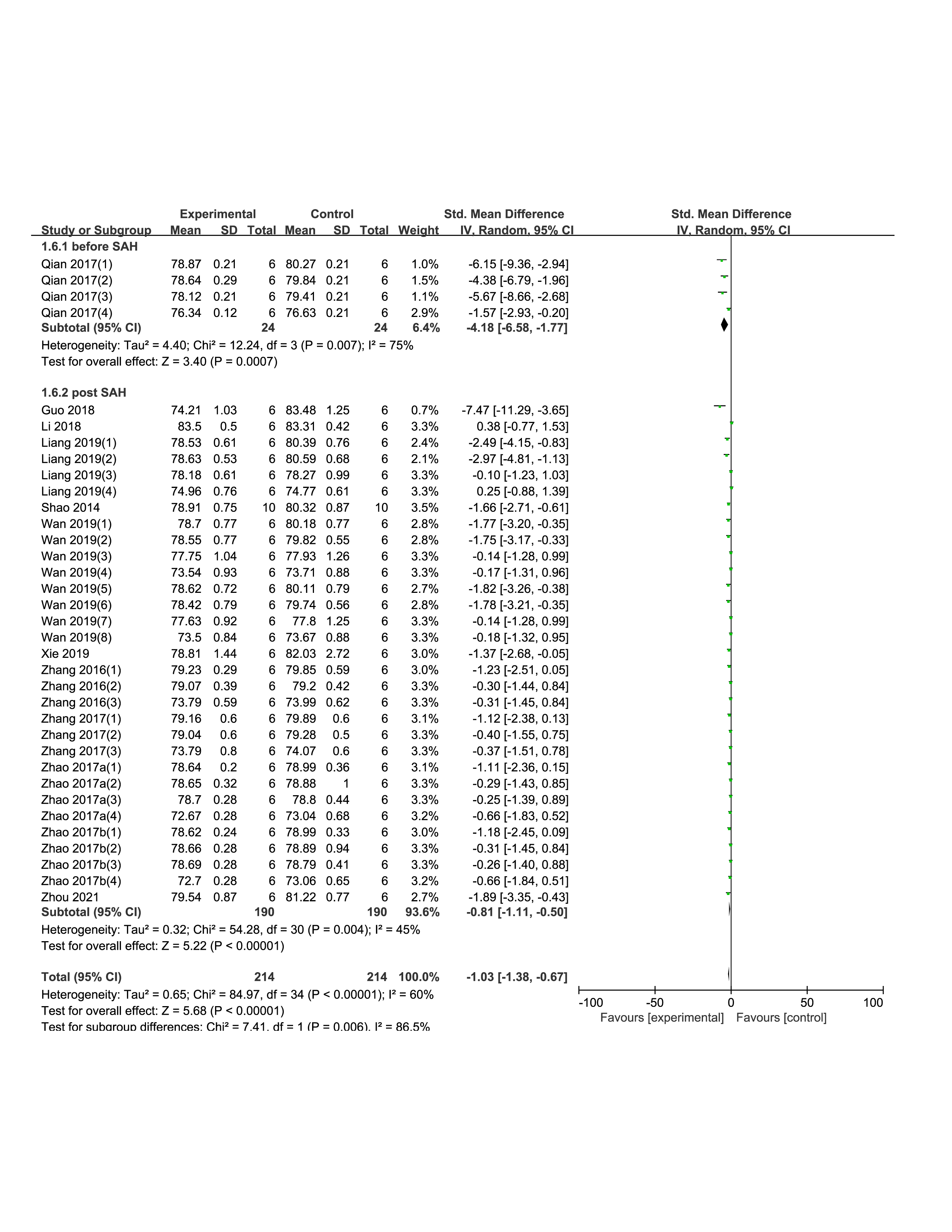

Supplement: Supplementary file 1 [file Image6.tif]

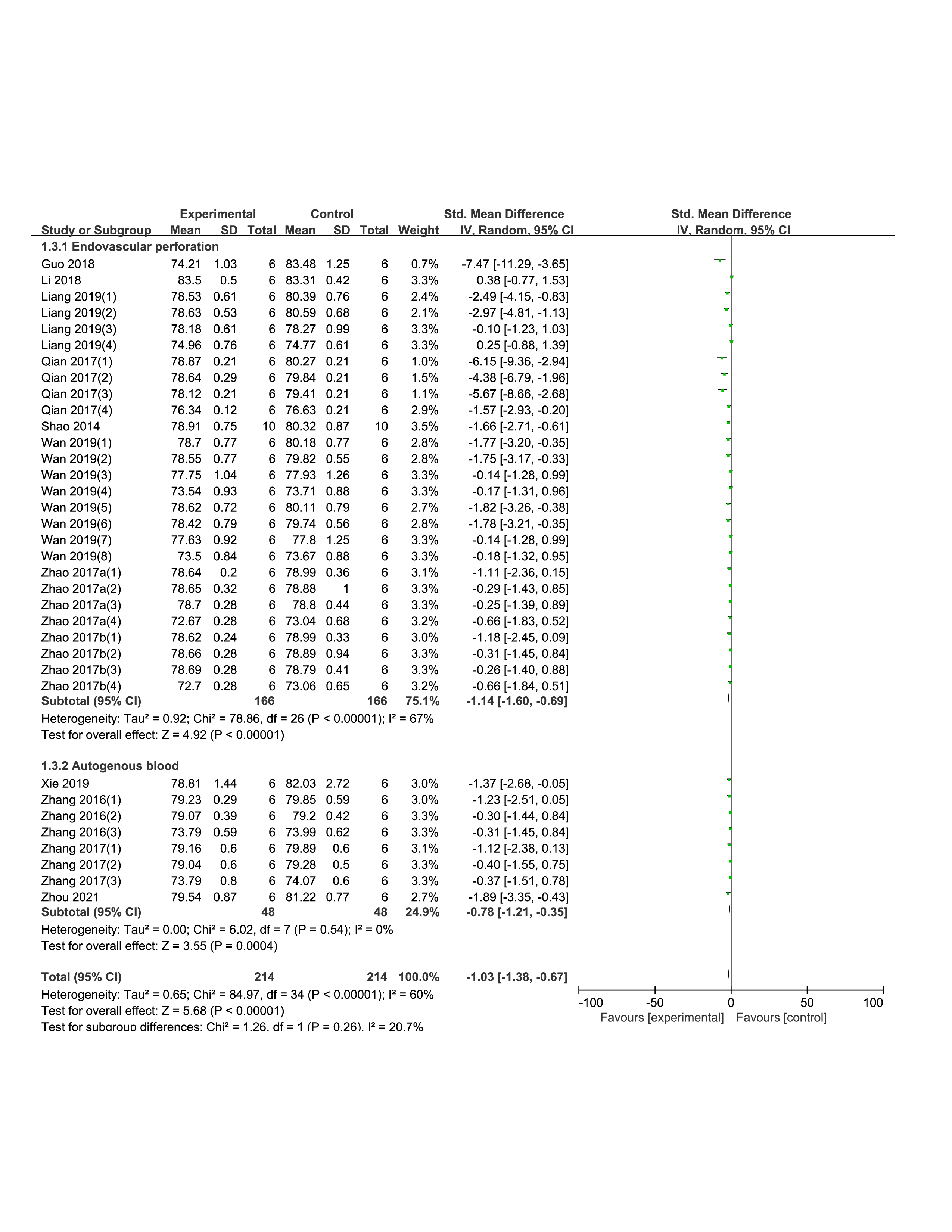

Supplement: Supplementary file 2 [file Image3.tif]

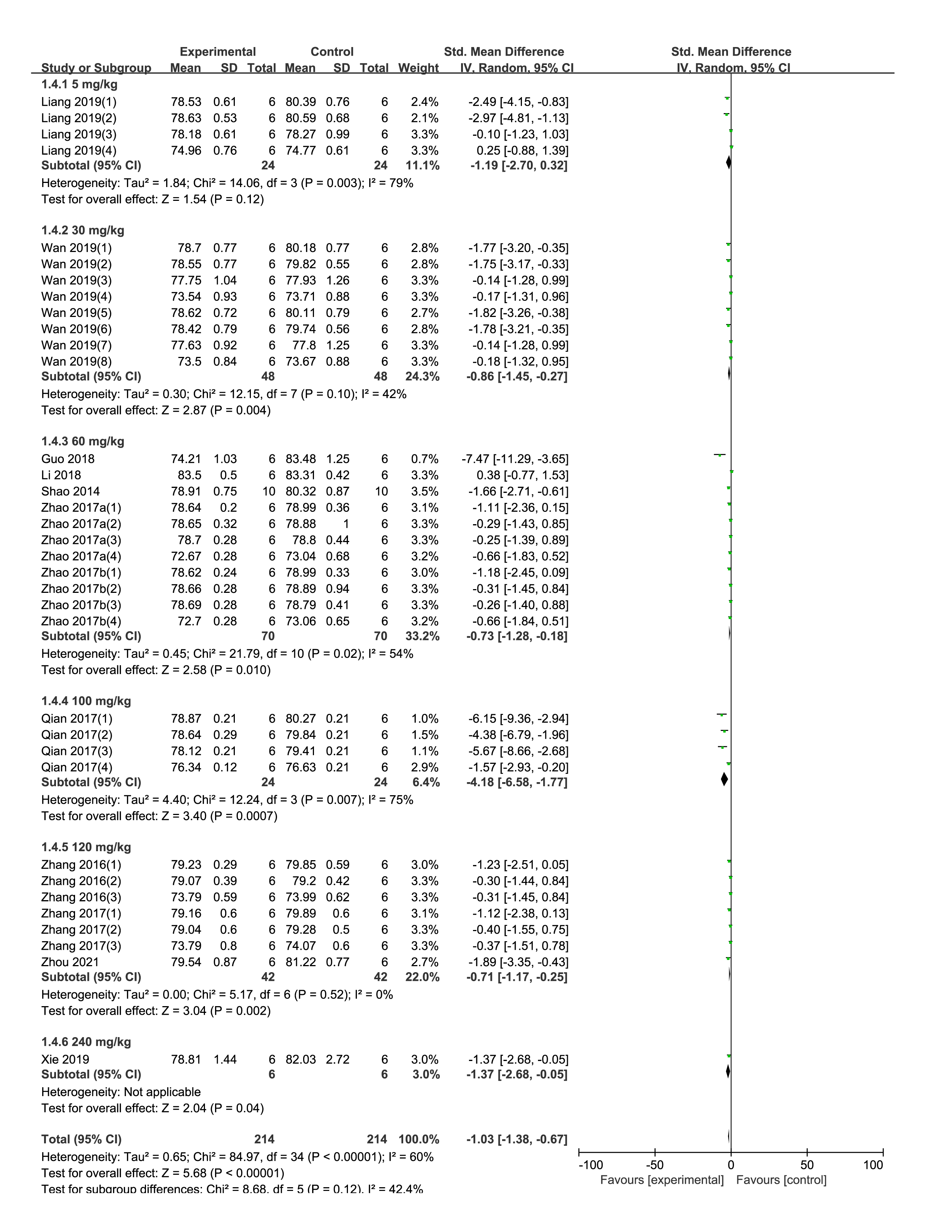

Supplement: Supplementary file 3 [file Image4.tif]

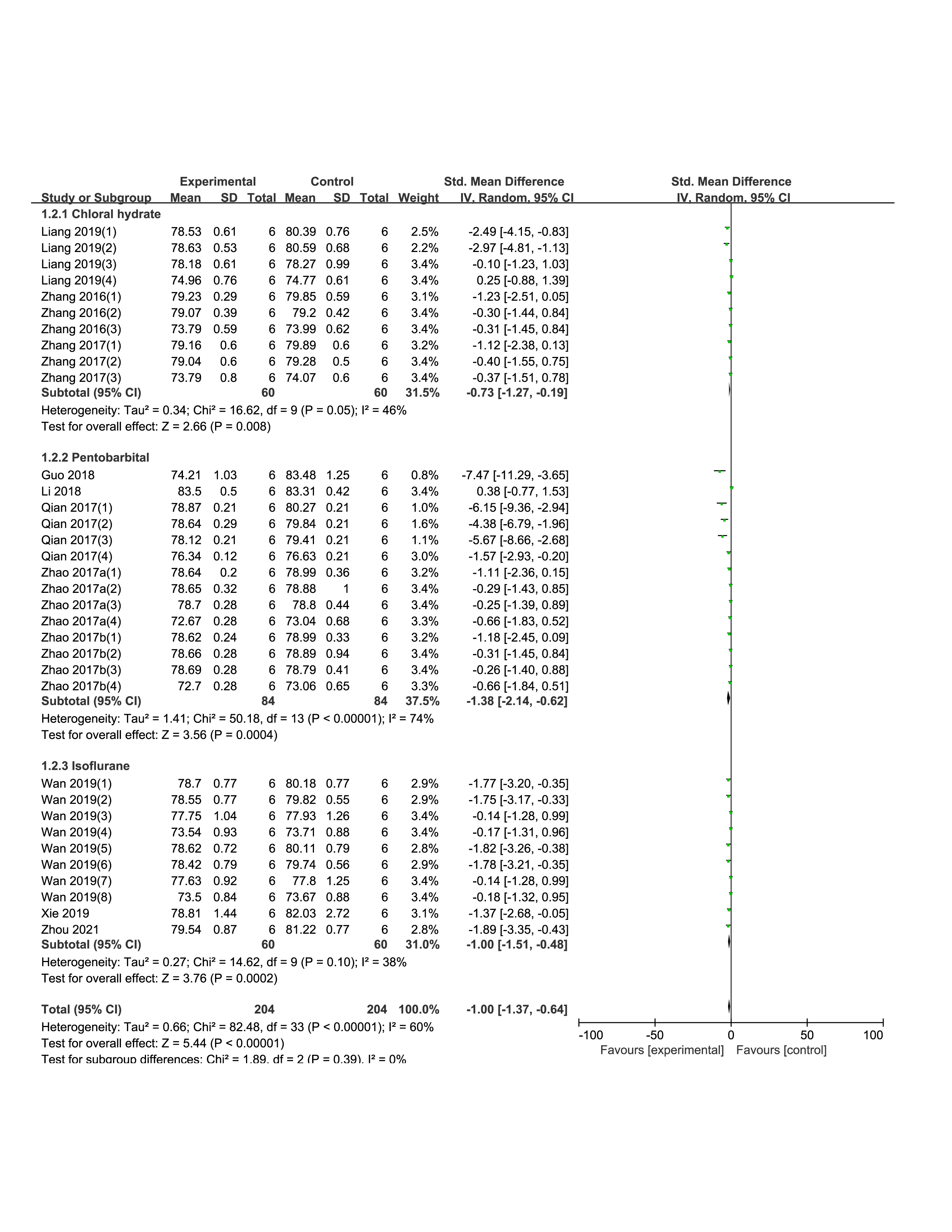

Supplement: Supplementary file 4 [file Image2.tif]

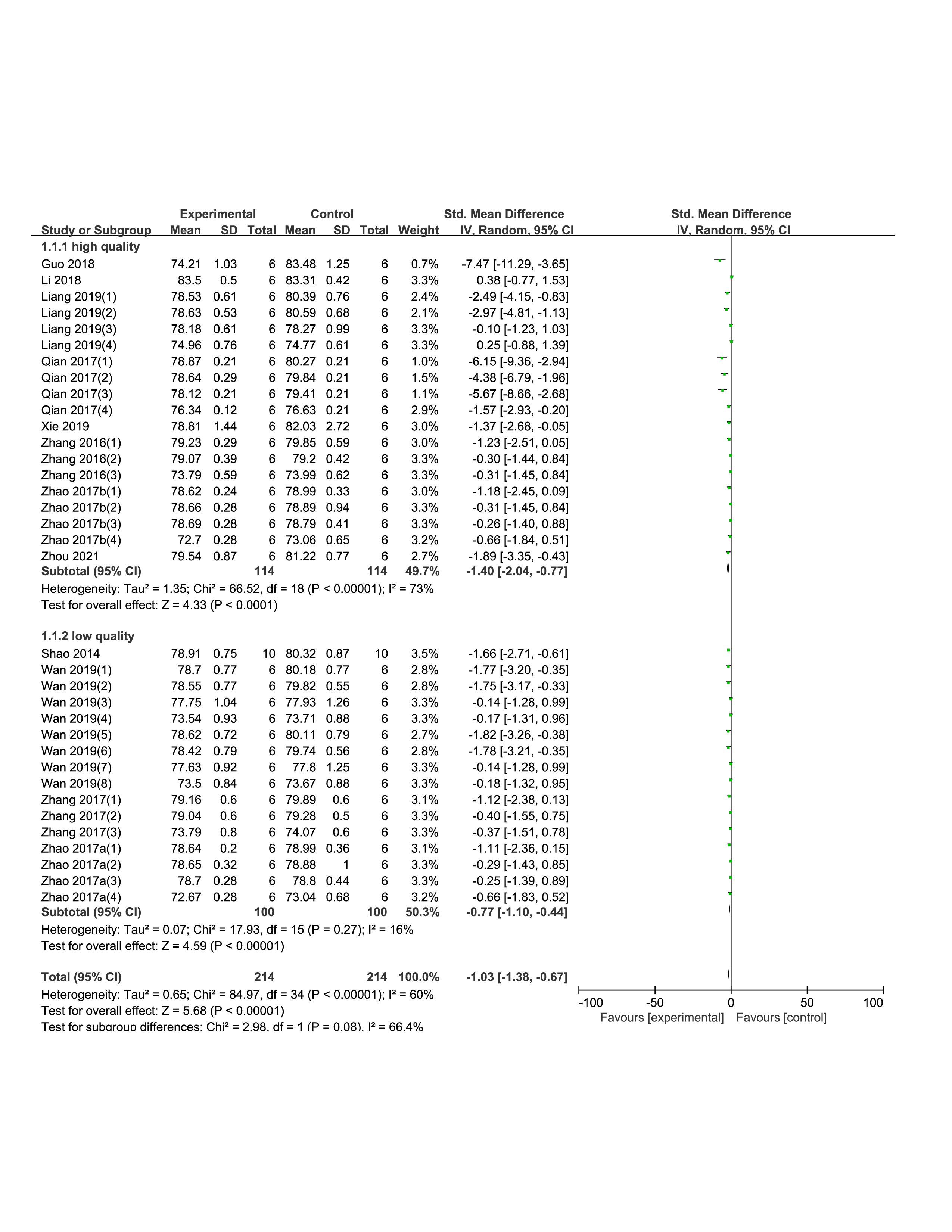

Supplement: Supplementary file 5 [file Image1.tif]

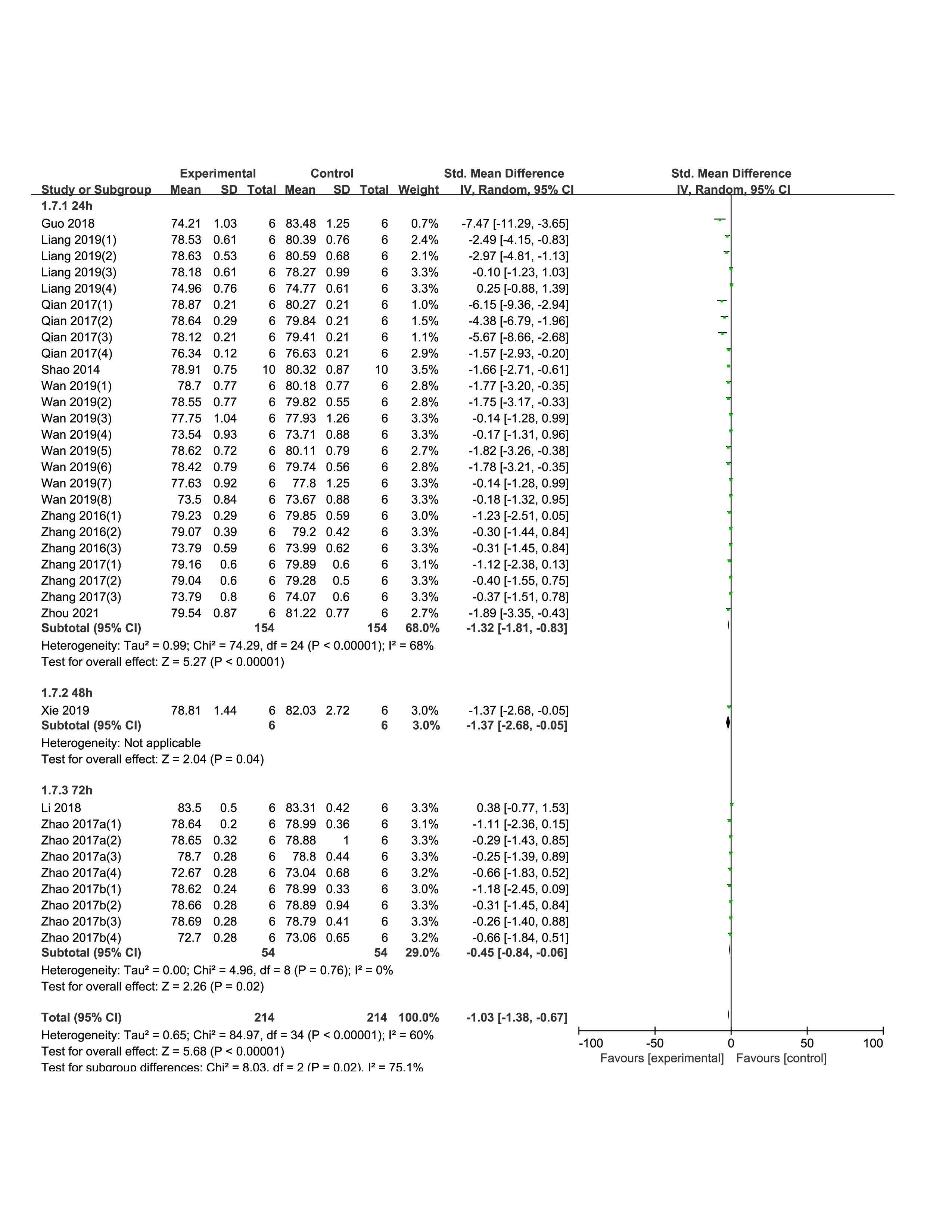

Supplement: Supplementary file 6 [file Image7.tif]

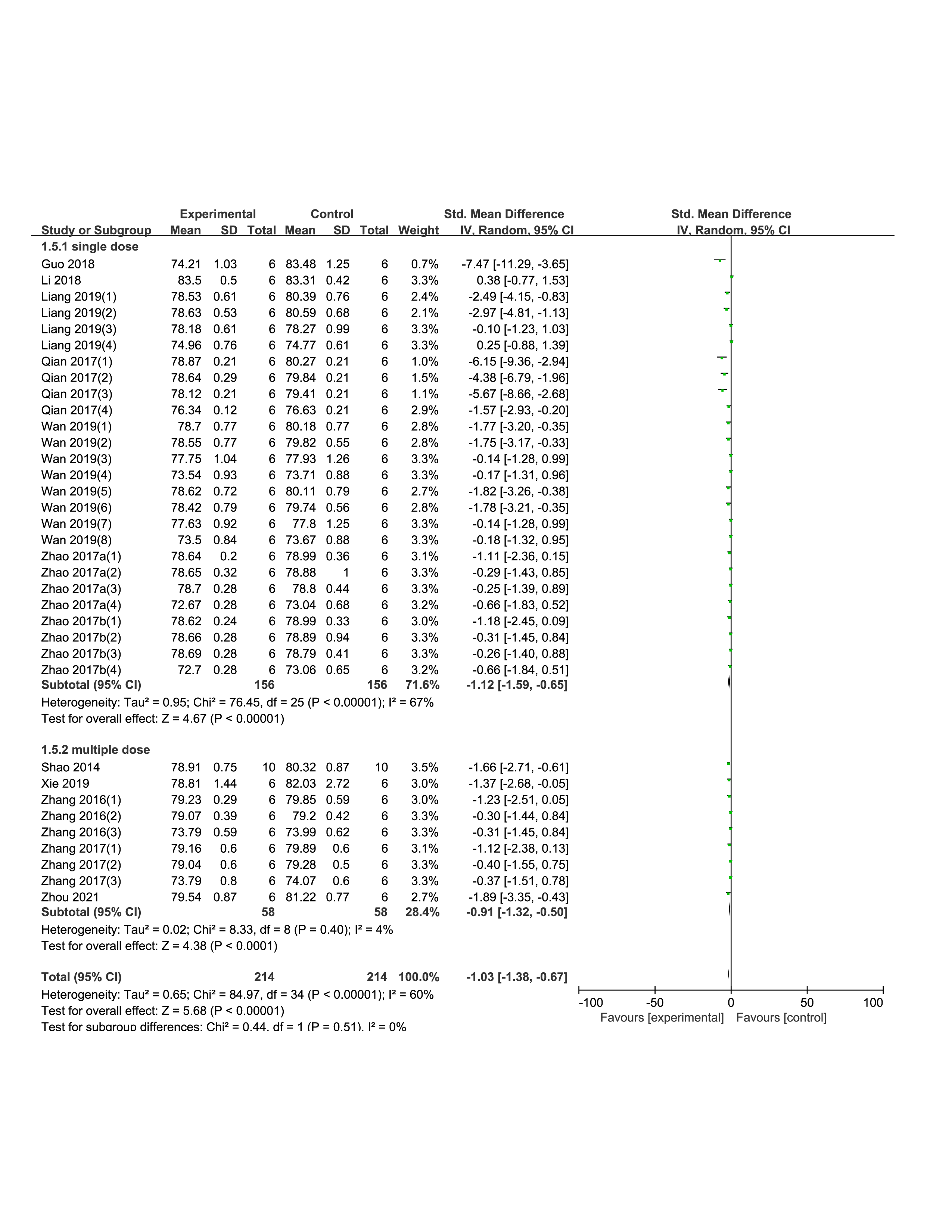

Supplement: Supplementary file 7 [file Image5.tif]
